# Comparing pedestal structure in JET-ILW H-mode plasmas with a model for stiff ETG turbulent heat transport

**DOI:** 10.1098/rsta.2021.0228

**Published:** 2023-02-20

**Authors:** A. R. Field, B. Chapman-Oplopoiou, J. W. Connor, L. Frassinetti, D. R. Hatch, C. M. Roach, S. Saarelma, JET contributors

**Affiliations:** ^1^ United Kingdom Atomic Energy Authority, Culham Centre for Fusion Energy, Culham Science Centre, Abingdon, Oxon OX14 3DB, UK; ^2^ Division of Fusion Plasma Physics, KTH Royal Institute of Technology SE-100 44 Stockholm, Sweden; ^3^ Institute for Fusion Studies, University of Texas at Austin, Austin, TX 78712, USA

**Keywords:** pedestal, H-mode, heat transport, stiffness, turbulence, ETG

## Abstract

A predictive model for the electron temperature profile of the H-mode pedestal is described, and its results are compared with the pedestal structure of JET-ILW plasmas. The model is based on a scaling for the gyro-Bohm normalized, turbulent electron heat flux $q_{e}/q_{e,\text{gB}}$ resulting from electron temperature gradient (ETG) turbulence, derived from results of nonlinear gyrokinetic (GK) calculations for the steep gradient region. By using the local temperature gradient scale length $L_{T_{e}}$ in the normalization, the dependence of $q_{e}/q_{e,\text{gB}}$ on the normalized gradients $R/L_{T_{e}}$ and $R/L_{n_{e}}$ can be represented by a unified scaling with the parameter $\eta_{e}=L_{n_{e}}/L_{T_{e}}$, to which the linear stability of ETG turbulence is sensitive when the density gradient is sufficiently steep. For a prescribed density profile, the value of $R/L_{T_{e}}$ determined from this scaling, required to maintain a constant electron heat flux $q_{e}$ across the pedestal, is used to calculate the temperature profile. Reasonable agreement with measurements is found for different cases, the model providing an explanation of the relative widths and shifts of the $T_{e}$ and $n_{e}$ profiles, as well as highlighting the importance of the separatrix boundary conditions. Other cases showing disagreement indicate conditions where other branches of turbulence might dominate.

This article is part of a discussion meeting issue ‘H-mode transition and pedestal studies in fusion plasmas’.

## 1. Introduction

The enhanced energy confinement of tokamak H-mode plasmas [1] is believed to result from E×B shear flow suppression of ion scale turbulence ($k_{y}\rho_{i}∼O(1)$, where $k_{y}$ is the wave number perpendicular to the flux surfaces and to the magnetic field B and $\rho_{i}$ is the ion Larmor radius) [2] within a localized edge transport barrier (ETB) referred to as the pedestal, which forms just inside the last-closed flux surface (LCFS). The radial electric field within the ETB is proportional to the ion pressure gradient $E_{r}∼p_{i}^{\prime }/(enB)$ (where ${\;}^{\prime }=d/dr$ and r is the minor radius) [3] and $p^{\prime }$ is maintained by the residual, conducted heat flux $q_{\text{cond}}$ across the pedestal remaining after accounting for radiation and energy losses due to edge-localized modes (ELMs)^1^ [4].

The predictive EPED model [5] for the total pressure at the pedestal top $p_{\text{ped}}$ assumes that the pressure pedestal width $\Delta_{p}$ is determined by the stability of kinetic ballooning modes (KBMs), which limit $p^{\prime }$, yielding the relation $\Delta_{p}\propto \beta_{p}^{1/2}$, where $\beta_{p}$ is the pedestal pressure normalized to the energy density of the poloidal magnetic field.^2^ The pedestal height is determined by increasing $p_{\text{ped}}$ until the MHD stability limit set by peeling-ballooning instabilities [6] is reached, above which an ELM would be triggered. To determine the electron temperature at the pedestal top $T_{e,\text{ped}}$, which is required as a boundary condition for modelling the core temperature profiles, it is hence necessary to assume a prescribed pedestal density $n_{e,\text{ped}}$.

Typically, equal electron and ion temperatures ($T_{e}=T_{i}$) and equal widths for the electron density, temperature and pressure pedestals ($\Delta_{n_{e}}=\Delta_{T_{e}}=\Delta_{p}$) are assumed, which limits the veracity of predictions made using EPED. To improve the model, it is desirable to be able to predict the $T_{e}$ *profile* given a prescribed density profile, which would obviate the necessity to assume equal temperature and density pedestal widths. Here, we present such a predictive model for the $T_{e}$ profile based on a model for ‘stiff’^3^ turbulent electron heat transport due to electron temperature gradient-driven (ETG) turbulence [7], which due to its fine spatial scale is not significantly affected by equilibrium E×B shear.

It was noted in ref. [4] that in JET-ILW (ITER-like-wall) pedestals, the parameter $\eta_{e}=L_{n_{e}}/L_{T_{e}}$, where the gradient scale length is defined as $L_{x}=x/x^{\prime }$, averaged across the steep-density gradient region of the pedestal, appears to saturate at values ${\left\langle \eta_{e}\right\rangle }_{\text{ped}}∼O(2)$ at high heating power. Such observations have been made on various other tokamaks (see refs. [75–81] of ref. [8]). This value lies just above the linear stability threshold of ETG micro-instabilities of $\eta_{e}∼0.8$ [9], which is an indication that stiffness of the turbulent electron heat transport due to ETG turbulence may be limiting the $T_{e}$ gradient across the pedestal.

A similar predictive pedestal model for the pedestal $T_{e}$ profile to that presented here is discussed in ref. [8], which is based on a scaling for the turbulent electron heat diffusivity $\chi_{e}$ derived from nonlinear GK calculations using the GK code CGYRO [10] for the steep-density gradient region of several different DIII-D pedestals. The scaling $\chi_{e}$ with $\eta_{e}$ and the normalized temperature gradient $R/L_{T_{e}}$ (where R is the major radius) proposed in ref. [8] is shown in ref. [11] to be consistent with that derived in a similar study for JET-ILW pedestals, upon which the model presented here is based. This suggests that a common mechanism underlies the turbulent electron heat transport across the pedestals studied in both devices.

Note that these models, which are based on a critical $\eta_{e}$, appropriate for the steep-density gradient region, are not the only predictive models for the pedestal $T_{e}$ profile. Heuristic models exist which are based on assumptions consistent with observations. An example of such a model [4], which assumes a constant $\eta_{e}$ across the pedestal and infinite stiffness, i.e. $\eta_{e}$ clamped at $\eta_{e,\text{cr}}$, is discussed in §5. An alternative model is that of Luda *et al.* [12], which is based on observations that the parameter $T_{e,\text{ped}}/\langle T_{e,\text{ped}}^{\prime }\rangle ∼2\;\mathrm{cm}$ (where $\left\langle T_{e,\text{ped}}^{\prime }\right\rangle$ is the average pedestal $T_{e}$ gradient) has been found to be relatively constant for a subset of pedestals on several devices [13]. This is then used in a transport model, together with an assumed pedestal width, to determine the heat diffusivity $\chi_{e}$ across the pedestal that satisfies this condition.

The justification for this model discussed in ref. [12] is that this normalized temperature gradient $R/L_{T_{e}}$, averaged over the pedestal, *might be interpreted as the drive for turbulent transport, and therefore can be associated with electron temperature gradient (ETG) modes or micro-tearing modes (MTMs)*. However, from the earlier discussion and refs [8,11], we learn that in the steep-density gradient region, ETG turbulence exhibits a threshold $\eta_{e,\text{cr}}$ rather than a threshold $R/L_{T_{e},\text{cr}}$. Also, as can be seen in the JET-ILW pedestal profiles shown in the following figures, $R/L_{T_{e}}$ varies considerably across the pedestal, so the electron heat transport is not governed by a constant critical value of this parameter.

The threshold behaviour of ETG turbulence is dependent on the magnitude of the normalized density gradient $R/L_{n_{e}}$. In the steep-density gradient region of the pedestal, where $R/L_{n_{e}}∼O(10-100)$, the critical temperature gradient $R/L_{T_{e},\text{cr}}\propto R/L_{n_{e}}$; hence, there is a critical $\eta_{e,\text{cr}}$ for the finite growth rate. However, as discussed in ref. [9], when the density gradient is weak, e.g. inside the top of the pedestal where $R/L_{n_{e}}∼O(1-10)$, $R/L_{T_{e},\text{cr}}$ is expected to be independent of $R/L_{n_{e}}$ and to be a function of other parameters, e.g. $R/L_{T_{e},\text{cr}}(\hat{s}/q,\tau ,\kappa ,\epsilon ,\ldots )$, where the magnetic shear $\hat{s}=rq^{\prime }/q$, $\tau =Z_{\text{eff}}T_{e}/T_{i}$, κ is the flux-surface elongation and the inverse aspect ratio ϵ=r/R.

These different threshold behaviours of ETG turbulence reflect the different dynamics in the presence of a strong or weak density gradient. The first case with the critical $\eta_{e,\text{cr}}$ corresponds to the ‘slab’ branch when the parallel resonance ($\omega ∼v_{\text{th},e}k_{∥}$, where $v_{\text{th},e}$ is the electron thermal velocity and $k_{∥}$ is the parallel wave number) dominates the dynamics, while the second corresponds to the ‘toroidal’ branch when cross-field (curvature and grad-B) drifts dominate [14]. In the GENE simulations for the steep-density gradient region of JET-ILW pedestals discussed in ref. [11], upon the results of which this work is based, an increasing contribution of high-$k_{∥}$ slab modes to the heat flux is observed when $R/L_{T_{e}}$ is large and the ETG turbulence is driven hard. Toroidal ETG modes, with a fine radial scale ($k_{x}\gg k_{y}$, where $k_{x}$ is the radial wave number), are also found to be unstable and to peak in amplitude off the outboard mid-plane [11,15], as well as some KBMs just inside the LCFS.

Other studies have shown ETG modes to be dominant in the steep gradient region of JET-ILW pedestals, e.g. in ref. [16], it is shown that ETG turbulence conducts ∼80% of the conducted power in the electron channel. In ref. [15], it is shown that for a particular JET-ILW equilibrium, similar to that of the 1.4 MA pulses discussed in §4a, the dominant modes are a novel type of toroidal ETG mode, driven far from the mid-plane, with a large spatial scale ($k_{y}\rho_{i}∼O(1)$).

The remainder of this article is structured as follows: in §2, the underlying physics of the model presented here is explained, which is based on a scaling of the locally gyro-Bohm normalized, turbulent electron heat flux with $\eta_{e}$, calculated using the actual, local $L_{T_{e}}$ in the pedestal. This scaling can then be used for numerical integration of the pedestal $T_{e}$ profile for a prescribed $n_{e}$ profile, as described in §3. In §4, this method is used to calculate the pedestal $T_{e}$ profile, and results are compared with measured pedestal profiles for several different JET-ILW pulses. A simple analytic model of the pedestal $T_{e}$ profile based on a constant $\eta_{e,\text{cr}}$ is discussed in §5, as is an interesting case when the numerical model fails, for which an alternative heat flux scaling is proposed. Finally, the conclusions of this study and outlook for further work are presented in §6.

## 2. The ETG heat flux manifold

Recently, nonlinear GK simulations for JET-ILW H-mode plasmas using the GK code GENE [17] have been used to quantify the stiffness of the saturated, turbulent electron heat flux $q_{e}$ in the steep-density gradient region of the pedestal. These calculations, described in ref. [11], include a detailed examination of the turbulent spectra by means of linear GK simulations. In brief, the nonlinear calculations performed to quantify the dependence of the turbulent electron heat flux on the electron temperature and density gradients in the steep density gradient region of the pedestal were local, electron scale GK simulations, with two dynamic species (electrons and deuterons, assuming $T_{i}=T_{e}$), with the effect of impurities included by the effect of $Z_{\text{eff}}$ on collisions alone. For details of these calculations and of the predicted turbulent spectra, the reader is referred to ref. [11].

A set of simulations were run in which the normalized gradients of temperature $R/L_{T_{e}}$ and density $R/L_{n_{e}}$ were scanned independently around the nominal experimental value, holding the corresponding parameter fixed. The resulting electron heat flux $q_{e}$ normalized to a constant gyro-Bohm heat flux $q_{e,\text{gB}}$ was found to scale as $q_{e}/q_{e,\text{gB}}\propto {\left(R/L_{n_{e}}\right)}^{-1}$ for the $R/L_{n_{e}}$ scan and $\propto {\left(R/L_{T_{e}}-R/L_{T_{e},\text{cr}}\right)}^{3}$ for the $R/L_{T_{e}}$ scan. Here, $q_{e,\text{gB}}$ is calculated using the nominal experimental parameters and is defined using the major radius R as the gradient scale length: $q_{e,\text{gB}}=n_{e}\chi_{e,gB}T_{e}/R,$ where $\chi_{e,gB}=v_{\text{th},e}\rho_{e}^{2}/R$, $v_{\text{th},e}$ is the electron thermal velocity and $\rho_{e}$ is the electron Larmor radius. For slab-ETG modes, the critical normalized temperature gradient is proportional to that of the density, i.e. $R/L_{T_{e},\text{cr}}=\eta_{e,\text{cr}}R/L_{n_{e}}$, where the linear stability threshold $\eta_{e,\text{cr}}∼0.8$ [9].

Similar nonlinear simulations using the CGYRO GK code [10] have been used to determine the scaling of the electron heat flux in the steep gradient region of DIII-D H-mode pedestals [8]. The resulting scaling of $\chi_{e}/\chi_{e,gB}$ with $\eta_{e}$ is used in a numerical pedestal model to compute the $T_{e}$ profile. By using the actual, local value of $L_{T_{e}}$ in each simulation to calculate the gyro-Bohm normalization rather than the fixed scale length R, the resulting scaling (for six different cases, at three radial locations in two different pulses) could be approximated by the linear relation:

2.1 $$\chi_{e}=\alpha (\eta_{e}-\eta_{e,\text{cr}})\left(\frac{v_{\text{th},e}\rho_{e}^{2}}{L_{T_{e}}}\right)\equiv \alpha (\eta_{e}-\eta_{e,\text{cr}})\chi_{e,\text{MgB}}$$

with the fitted constant α∼1.5 and nonlinear threshold $\eta_{e,\text{cr}}∼1.4$. Here, we have introduced the modified gyro-Bohm diffusivity defined using the local $L_{T_{e}}$ as $\chi_{e,\text{MgB}}=\chi_{e,gB}(R/L_{T_{e}})$. Similarly, the local gyro-Bohm heat flux defined using the local $L_{T_{e}}$ is referred to here as $q_{e,\text{MgB}}=q_{e,\text{gB}}{\left(R/L_{T_{e}}\right)}^{2}$, as shown in ref. [11].

Remarkably, in ref. [11], it is shown that the results of these two separate studies can be represented by the same, approximate linear scaling in $\eta_{e}$, with nearly the same fit coefficients α and $\eta_{e,\text{cr}}$. In the JET-ILW study, gradients scans were performed for the same two 1.4 MA H-mode pulses with 16 MW of heating power with ‘low’ and ‘high’ rates of gas fuelling for which pedestal profiles are shown in figure 2.

In the following, $Q_{e}^{⋆}=q_{e}/q_{e,\text{MgB}}$ denotes the electron heat flux normalized to the modified gyro-Bohm heat flux. As reported in ref. [11], fits of the turbulent heat flux data from these GENE scans to a linear scaling for $Q_{e}^{⋆}$ of the form:

2.2 $$Q_{e}^{⋆}\equiv \frac{q_{e}}{q_{e,\text{MgB}}}\equiv \frac{\chi_{e}}{\chi_{e,\text{MgB}}}=\alpha (\eta_{e}-\eta_{e,\text{cr}})$$

which follows from equation (2.1), gave values of α=1.19 and $\eta_{e,\text{cr}}=1.49$ for the scans at low fuelling rate and α=1.7 and $\eta_{e,\text{cr}}=1.9$ for the scans at high fuelling rate, while a fit to both data sets together yielded α=1.74 and $\eta_{e,\text{cr}}=1.81$.

Note that a nonlinear fit of the form:

2.3 $$Q_{e}^{⋆}=\alpha {\left(\eta_{e}-\eta_{e,\text{cr}}\right)}^{\beta }$$

was found in ref. [11] to better represent the data from both data sets with α=0.85, β∼1.43 and $\eta_{e,\text{cr}}=1.28$, i.e. with a somewhat stronger than linear dependence on $\eta_{e}-\eta_{e,\text{cr}}$. In the analytic model presented later, the linear form in $\eta_{e}$ is used as this is algebraically tractable. The results from this analytic model can then be used to provide an initial estimate of $R/L_{T_{e}}$ as input to an iterative, numerical algorithm used to solve the nonlinear scaling equation (2.3) for $R/L_{T_{e}}$.

It is straightforward to show how the $q_{e}$ dependencies for the scans of $R/L_{n_{e}}$ and $R/L_{T_{e}}$ found in ref. [11] and discussed earlier are consistent with equation (2.1), at least in the limit that $\eta_{e}\gg \eta_{e,\text{cr}}$. equation (2.2) can be expressed in terms of $R/L_{T_{e}}$ and $R/L_{n_{e}}$ as follows:

2.4 $$\frac{q_{e}}{q_{e,\text{gB}}}=\alpha {\left(\frac{R}{L_{n_{e}}}\right)}^{-1}\left(\frac{R}{L_{T_{e}}}-\frac{R}{L_{T_{e},\text{cr}}}\right){\left(\frac{R}{L_{T_{e}}}\right)}^{2},$$

where the nonlinear threshold $R/L_{T_{e},\text{cr}}=\eta_{e,\text{cr}}(R/L_{n_{e}})$. This relation encapsulates both the inverse dependence of $q_{e}/q_{e,\text{gB}}$ on $R/L_{n_{e}}$ and, in the limit that $R/L_{T_{e}}\gg R/L_{T_{e},\text{cr}}$, its cubic dependence on $R/L_{T_{e}}$. It is a theoretical prediction that, far above threshold, the turbulent heat flux of critically balanced, saturated turbulence should scale as $q_{e}/q_{e,\text{gB}}\propto {\left(R/L_{T_{e}}\right)}^{3}$ [18].

**Figure 1.**
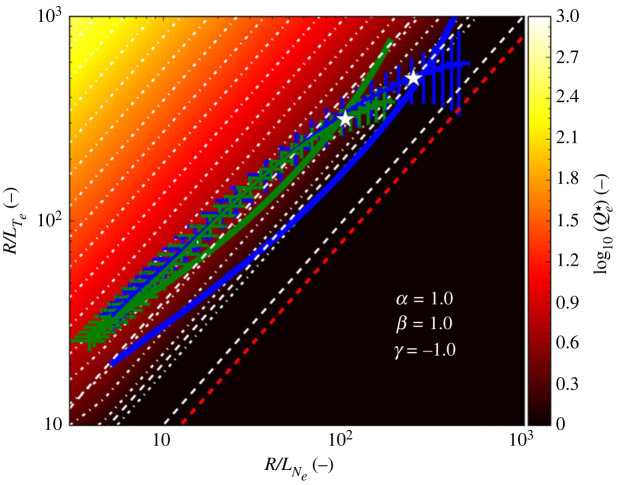
The normalized electron heat flux manifold $Q_{e}^{⋆}(R/L_{n_{e}},R/L_{T_{e}})$ described by equation (2.5) assuming α=β=1 and γ=−1. The diagonal (white-dashed) lines correspond to $\eta_{e}=1,2,4$ (bottom-top) and the contours (white-dotted) are at constant $Q_{e}^{⋆}$. The linear ETG stability threshold $R/L_{T_{e},\text{cr}}=0.8R/L_{n_{e}}$ from [9] is also shown (red dashed). The lines with uncertainties show experimental loci $\left(R/L_{n_{e}},R/L_{T_{e}}\right)$ of the two sets of pedestal profiles shown in figure 2 for JET-ILW 1.4 MA H-mode pulses at 16 MW of heating with low (blue) and high (green) rates of gas fuelling together with the corresponding predictions of the stiff ETG model (solid lines). Note that the white stars indicate the mid-pedestal location for which the GENE simulations were performed. (Online version in colour.)

A relation such as equation (2.4) can be referred to as a heat flux ‘manifold’, i.e. in this case the surface $Q_{e}^{⋆}(R/L_{n_{e}},R/L_{n_{e}})$. Such a manifold is shown in figure 1, in this case described by the relation:

2.5 $$Q_{e}^{⋆}=\alpha {\left(\frac{R}{L_{T_{e}}}-\frac{R}{L_{T_{e},\text{cr}}}\right)}^{\beta }{\left(\frac{R}{L_{n_{e}}}\right)}^{\gamma }.$$

With the parameters β=1 and γ=−1 and $R/L_{T_{e},\text{cr}}=\eta_{e,\text{cr}}R/L_{n_{e}}$, this form is equivalent to equation (2.4). In figure 1, the linear threshold $R/L_{T_{e},\text{cr}}$ for ETG turbulence from ref. [9] is used which is equivalent to assuming $\eta_{e,\text{cr}}=0.8$ at sufficiently high values of $R/L_{n_{e}}$ that the slab branch of ETG turbulence is prevalent, as is appropriate for the density steep gradient region of the pedestal. Note that on this manifold, contours of constant $\eta_{e}$, which are diagonal lines in ($\log(R/L_{n_{e}}),\log(R/L_{T_{e}})$) space are also lines of constant $Q_{e}^{⋆}$. Note that these are not necessarily contours of the absolute heat flux $q_{e}$ because $Q_{e}^{⋆}\propto q_{e}/(n_{e}T_{e}^{1/2}T_{e}^{\prime 2})$.

The heat flux manifold shown in figure 1 is shown overlaid by the experimental loci, i.e. the trajectory formed by pairs of values $\left(R/L_{n_{e}},R/L_{T_{e}}\right)$ across the pedestal, determined from the pre-ELM pedestal profiles for two 1.4 MA/1.7 T JET-ILW D pulses (#84794 and #87342) with similar heating powers of 16 & 14 MW,  respectively, but with low and high rates of gas fuelling, i.e. $\Gamma_{D2}=0.3$ and $1.8\times 10^{22}\;e\;s^{-1}$, from which it can be seen that these loci *approximately* follow contours of constant $\eta_{e}∼2-4$.

This behaviour can be understood as follows. As the turbulent heat transport is stiff, i.e. approximately $q_{e}\propto {\left(R/L_{T_{e}}-R/L_{T_{e},\text{cr}}\right)}^{3}$, we may expect the temperature gradient $T_{e}^{\prime }$ to adjust such that the absolute electron heat flux $q_{e}$ remains constant across the pedestal (as would be expected with minimal sources and sinks in the pedestal), with the resulting profiles ensuring that the locally gyro-Bohm normalized heat flux $Q_{e}^{⋆}$ follows approximately contours of constant $\eta_{e}$ not far above the threshold $\eta_{e,\text{cr}}$ for the onset of turbulence. In other words, when $\eta_{e}-\eta_{e,\text{cr}}>O(1)$, i.e. at values of $\eta_{e}∼O(2)$ where $Q_{e}^{⋆}∼O(1)$, the absolute heat flux increases rapidly with $R/L_{T_{e}}$, hence clamping the experimental $\left(R/L_{n_{e}},R/L_{T_{e}}\right)$ loci to contours of approximately constant $\eta_{e}∼O(2)$. It is shown in §3 how this property, embodied in equation (2.4), can be used to predict the temperature profile for a prescribed density profile and boundary conditions at the separatrix.

## 3. Numerical model for $T_{e}$ profile

At each flux surface across the pedestal of area S, the electron temperature gradient $T_{e}^{\prime }$ will adjust such that the ETG turbulence conducts the imposed heat flux from the plasma interior, i.e. $q_{e}=P_{e,\text{cond}}/S$, where $P_{e,\text{cond}}$ is the conducted electron loss power. For simplicity, energy losses from the electrons due to ionization, radiation and collisional exchange with the ions are neglected.^4^ Hence, $P_{e,\text{cond}}$ is assumed to be the same as the electron loss power $P_{e,\text{sep}}$ crossing the LCFS. Furthermore, the relatively small fractional change in the flux surface area across the narrow pedestal region is neglected, i.e. $q_{e}=P_{e,\text{sep}}/S_{\text{sep}}$, where $S_{\text{sep}}$ is the area of the LCFS.^5^

If we assume that the turbulent electron heat flux obeys the scaling given by equation (2.4), to calculate the $T_{e}$ profile by numerical integration, it is necessary to solve this cubic equation in $R/L_{T_{e}}$ at each flux surface. Note that we expect this relation to be appropriate for the steep gradient region of the pedestal, for which the nonlinear GK calculations were performed, but we might expect departures from this scaling, e.g. inside the density pedestal top, where the density gradient is weaker and the electron scale turbulence has different characteristics.

The scaling given by equation (2.4) can be expressed as follows:

3.1 $${\left(\frac{R}{L_{n_{e}}}\right)}^{-1}{\left(\frac{R}{L_{T_{e}}}\right)}^{3}-\eta_{e,\text{cr}}{\left(\frac{R}{L_{T_{e}}}\right)}^{2}-\frac{q_{e}}{\left(\alpha q_{e,\text{gB}}\right)}=0,$$

i.e as a cubic polynomial $ax^{3}+bx^{2}+cx+d=0$ in $x=R/L_{T_{e}}$, where $a={\left(R/L_{n_{e}}\right)}^{-1}$, $b=-\eta_{e,\text{cr}}$, c=0 and $d=-q_{e}/(\alpha q_{e,\text{gB}})$. As all of these quantities are positive definite, it is simple to determine whether the discriminant^6^ of the polynomial Δ<0, thus proving that there is only one real root, which is the case for the calculations presented here.

To calculate $q_{e,\text{gB}}=en_{e}T_{e}v_{\text{th},e}{\left(\rho_{e}/R\right)}^{2}$, with $v_{\text{th},e}={\left(2eT_{e}/m_{e}\right)}^{1/2}$, $\rho_{e}=v_{\text{th},e}/\Omega_{e}$ and the electron gyro frequency $\Omega_{e}=eB/m_{e}$, the parameters $T_{e}$, $n_{e},$ and B are required. For the coefficients of equation (3.1), the parameter $R/L_{n_{e}}$ from the prescribed $n_{e}$ profile, the electron heat flux $q_{e}$, and the coefficients of the ETG heat flux scaling α and $\eta_{e,\text{cr}}$ are required.

Numerical integration of the $T_{e}$ profile requires starting at the separatrix with prescribed values of $T_{e,\text{sep}}$ and $n_{e,\text{sep}}$, e.g. as determined from measurements or from a SOL plasma model, assuming a prescribed density profile from which the profile of $R/L_{n_{e}}$ is calculated, and a particular value of $q_{e}=P_{e,\text{sep}}/S_{\text{sep}}$ and then using these to solve equation (3.1) for $R/L_{T_{e}}$.

The value of $R/L_{T_{e}}$ at the particular flux surface is then used in an explicit, forward integration to calculate the temperature at the next integration step $T_{e}[i+1]$, starting at the separatrix, where $T_{e}[0]=T_{e,\text{sep}}$ by iteration of:

3.2 $$T_{e}[i+1]=T_{e}[i]\left(1+\left(\frac{R}{L_{T_{e}}}\right)\right)[i]\left(\frac{\delta R}{R[i]}\right),$$

where [i] is the ith radial element of a vector and δR is the radial integration increment. A fixed value of $T_{e}$ at the separatrix is often assumed for JET-ILW H-mode plasmas of $T_{e,\text{sep}}∼100\;\mathrm{eV}$. This is justified because $T_{e,\text{sep}}$ is a weak function of the loss power $P_{e,\text{sep}}$ [19].

Numerical solution of the nonlinear form of the heat flux scaling given by equation (2.3), i.e. $Q_{e}^{⋆}=\alpha \;{\left(\eta_{e}-\eta_{e,\text{cr}}\right)}^{\beta }$, has been implemented. This algorithm uses the analytic solution of equation (2.4) to provide an initial guess for $R/L_{T_{e}}$ (and hence $\eta_{e}$), which is then repeatedly incremented by a small fraction until the nonlinear scaling is satisfied. Unless otherwise stated, the model $T_{e}$ profiles presented in the figures here are calculated assuming this nonlinear form, using the nominal coefficients α=0.85, β=1.28, and β=1.43, as appropriate for the 1.4 MA, low and high-gas JET-ILW pulses discussed in §4a.

## 4. Comparison of predicted with measured pedestal $T_{e}$ profiles

As a first test of the model described in §3, predicted $T_{e}$ profiles for JET-ILW H-mode pulses with different rates of gas fuelling, plasma currents and heating powers are compared with the experimentally measured profiles. Note that, at 1.4 MA, low-triangularity (δ) pulses are the same as those used in ref. [11] to determine the turbulent heat flux scaling, and also including higher current 3.5 MA pulse at high heating power with quite different parameters allows a more stringent test of the predictive capability of the model. The parameters of the analyzed pulses are presented in table 1. Note that the loss power components during the inter-ELM periods due to radiation $P_{\text{Rad}}^{\text{iELM}}$, ELMs $\left\langle P_{\text{ELM}}\right\rangle$ and inter-ELM heat transport $P_{\text{sep}}^{\text{iELM}}$ are determined using the method described in ref. [4].

**Table 1.** Parameters of the JET-ILW pulses discussed in §4: plasma current $I_{p}$, toroidal field $B_{t}$, input power $P_{\mathrm{in}}$, $D_{2}$ gas fuelling rate $\Gamma_{D2}$, averaging period $t_{0}-t_{1}$ and the loss power components due to radiation, ELMs (time averaged) and inter-ELM heat transport. Note that pulse #96482 was fuelled by both gas puffing and cryogenic deuterium ELM pacing pellets, injected at a repetition rate of ∼35 Hz.

pulse	$I_{p}$	$B_{t}$	$P_{\mathrm{in}}$	$\Gamma_{D2}$	$t_{0}-t_{1}$	$P_{\mathrm{Rad}}^{\mathrm{iELM}}$	$\left\langle P_{\mathrm{ELM}}\right\rangle$	$P_{\mathrm{sep}}^{\mathrm{iELM}}$
#	(MA)	(T)	(MW)	($10^{22}\;e\;s^{-1}$)	(s)	(MW)	(MW)	(MW)
84794	1.4	1.7	16.0±0.3	0.3	5.0–6.0	3.8±0.2	3.4±0.2	6.2±0.7
87342	1.4	1.7	13.9±0.4	1.8	5.5–8.8	1.3±0.2	7.2±0.1	5.4±0.5
96482	3.5	3.3	32.1±0.1	2.1	9.5–10.5	16.0±0.1	5.3±0.2	10.9±0.3
94662	3.0	2.8	26.1±0.1	0.0	9.0–10.0	11.3±0.1	5.5±0.2	9.3±0.3

For the 1.4 MA high-gas pulse #87342 with ∼14 MW heating power, a high ELM frequency ($f_{\text{ELM}}∼O(100)\;\mathrm{Hz}$) prevented determination of $\left\langle P_{\text{ELM}}\right\rangle$ from changes in the plasma stored energy $W_{\text{pl}}$ determined from magnetic measurements as described in ref. [4], so the same fraction of ELM loss power to the total heating power of $\left\langle P_{\text{ELM}}\right\rangle /P_{\text{in}}∼0.52$ is assumed as in the lower power ∼5 MW pulse #87346 at the same fuelling rate for which $f_{\text{ELM}}$ was low enough to determine $\left\langle P_{\text{ELM}}\right\rangle$ reliably.^7^

### (a) 1.4 MA/1.7 T low-δ pulses at low and high fuelling rates

**Figure 2.**
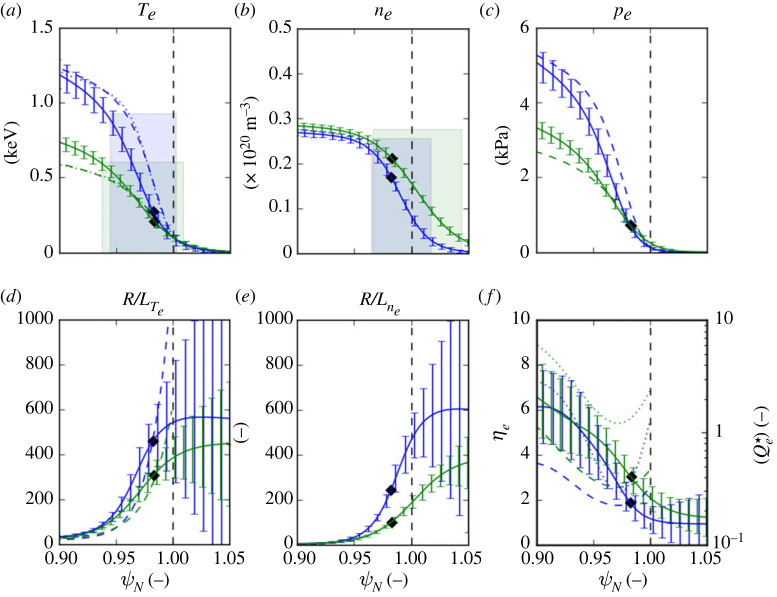
Pre-ELM averaged (∼80--100% of the inter-ELM period) pedestal profiles for two 1.4 MA JET-ILW H-mode pulses at low (#84794, blue) and high (#87342, green) rates of D gas fuelling with 16 and 14 MW of heating power, respectively, showing (with error bars): (*a*) electron temperature $T_{e}$, (*b*) density $n_{e}$, (*c*) pressure $p_{e}$, their normalized gradients (*d*) $R/L_{T_{e}}$, (*e*) $R/L_{n_{e}}$ and (*f*) the parameter $\eta_{e}$ (solid/dashed) and the locally gyro-Bohm normalized electron heat flux $Q_{e}^{⋆}$ (dotted) versus normalized poloidal flux $\psi_{N}$. The height, width and position of the mtanh() fits to the measured $T_{e}$ and $n_{e}$ profiles are indicated by the shaded bars. Profiles in (*a*, *c*, *d* and *f*) calculated using the stiff ETG model assuming the scaling: $Q_{e}^{⋆}=0.85{\left(\eta_{e}-1.28\right)}^{1.43}$ are shown dashed with mtanh() fits to the calculated profiles (dotted). The mid-pedestal locations at which the GENE calculations were performed are indicated by the filled diamond symbols. The uncertainties on the fitted profiles are obtained using a Monte-Carlo method using the uncertainty estimates on the fit parameters. (Online version in colour.)

Pedestal profiles for two 1.4 MA, low-δ H-mode pulses with low and high rates of $D_{2}$ gas fuelling (#84794 and #87342 at $\Gamma_{D2}∼0.3\&1.8\times 10^{22}\;e\;s^{-1}$, with 16 and 14 MW of heating power, respectively) [20,21] are shown in figure 2. The $T_{e}$ and $n_{e}$ profiles are mtanh( )  fits [22,23] to an ensemble of measured profiles from the high-resolution Thompson scattering system [24] from the pre-ELM phase of several inter-ELM periods, which are taken from the EUROfusion pedestal database [25]. Both profiles are shifted radially to ensure that $T_{e,\text{sep}}∼100\;\mathrm{eV}$, which is a typical value for JET-ILW [19] and mapped onto the normalized poloidal flux coordinate $\psi_{N}$ using a magnetic equilibrium reconstruction from EFIT [26].

Global linear GENE simulations presented in ref. [11] for these cases (in which $T_{i}=T_{e}$ was assumed) show that ion scale modes that would be responsible for any ion scale turbulent heat flux are largely suppressed by E×B flow shear and also that collisional, neo-classical ion heat transport accounts for ≲20% of the inter-ELM heat transport ($P_{i,\text{NC}}∼1.2\;\mathrm{MW}$ in #84794 and ∼0.6 MW in #87342). For calculation of the predicted $T_{e}$ profiles, it is assumed that the residual, conducted power across the pedestal during the inter-ELM periods is carried by turbulent electron heat transport, i.e. $P_{e,\text{sep}}=P_{\text{sep}}^{\text{iELM}}-P_{i,\text{NC}}$.

The effect of increasing the fuelling rate $\Gamma_{D2}$ between the two pulses shown in figure 2 by a factor ∼6 is to increase the separatrix density $n_{e,\text{sep}}$, while the pedestal density $n_{e,\text{ped}}$ remains largely unchanged, i.e. the relative separatrix density $n_{e,\text{sep}}/n_{e,\text{ped}}$ is approximately doubled. This increase reduces the normalized density gradient $R/L_{n_{e}}$ across the steep gradient region of the pedestal. Note that for both pulses the profile of $\eta_{e}$ increases from values ∼2–3 in the steep-density gradient to ∼6 at the top of the $T_{e}$ pedestal, i.e. there is a concomitant decrease of $R/L_{T_{e}}$ across the pedestal which partially compensates for the decrease of $R/L_{n_{e}}$ to maintain similar profiles of $\eta_{e}$. The result of reducing $R/L_{T_{e}}$ across the pedestal at a constant separatrix temperature $T_{e,\text{sep}}$ is to progressively decrease $T_{e}$ inwards across the pedestal, almost halving the pedestal top temperature $T_{e,\text{ped}}$.

Although the pedestal profiles look rather different for the two cases, the corresponding loci $\left(R/L_{n_{e}},R/L_{T_{e}}\right)$ shown in figure 1 almost overlay but importantly, the low-gas case (#84794) extends to higher values of $R/L_{n_{e}}$ and $R/L_{T_{e}}$ towards the separatrix. In the high-gas case, the effect of increasing $n_{e,\text{sep}}$ (and hence decreasing $R/L_{n_{e}}$ across the pedestal) is to reduce the values of $R/L_{T_{e}}$ required to maintain the normalized heat flux $Q_{e}^{⋆}$ corresponding to a constant absolute turbulent heat flux $q_{e}$ across the pedestal. At a given $\eta_{e}$, decreasing $n_{e}$ at the separatrix necessitates starting the integration of the $T_{e}$ profile with a higher gradient $T_{e}^{\prime }=\eta_{e}T_{e,\text{sep}}(n_{e}^{\prime }/n_{e,\text{sep}})$, this effect propagating inwards, increasing $T_{e}$ across the whole pedestal.

Pedestal parameters determined from the fitted profiles for the various cases are stated in table 2 and compared with similar parameters for the $T_{e}$ profiles calculated using the stiff ETG model in table 3. For the low-gas pulse #84794, $T_{e,\text{ped}}$ is close to the measured value (×0.97), while the $T_{e}$ pedestal width $\Delta_{T_{e}}$ is underpredicted (×0.65). This is because the values of $R/L_{T_{e}}$ required to satisfy the $Q_{e}^{⋆}$ scaling are too large outside the mid-pedestal location at which the GENE calculations were performed, hence increasing $T_{e}$ across the steep gradient region. However, this is compensated by too low a value of $R/L_{T_{e}}$ inside the mid-pedestal location, resulting overall in a reasonable prediction of $T_{e,\text{ped}}$ but a reduced pedestal width $\Delta_{T_{e}}$.

**Table 2.** Pedestal parameters of the JET-ILW pulses discussed in §4: $n_{e}$ and $T_{e}$ at pedestal top, pedestal widths $\Delta_{n,T}$ in $\psi_{N}$, relative shift $\delta_{n-T}$ of density and temperature pedestal positions and relative separatrix density.

pulse	$n_{e,\mathrm{ped}}$	$n_{e,\mathrm{sep}}/n_{e,\mathrm{ped}}$	$T_{e,\mathrm{ped}}$	$\Delta_{n_{e}}$	$\Delta_{T_{e}}/\Delta_{n_{e}}$	$\delta_{n-T}$
#	$\left(10^{20}\;m^{-3}\right)$	(−)	(keV)	($\psi_{N}$)	($\psi_{N}$)	($\psi_{N}$)
84794	0.26±0.01	0.33±0.05	0.93±0.05	0.05±0.01	0.57±0.06	0.017±0.003
87342	0.28±0.01	0.57±0.03	0.61±0.03	0.08±0.01	0.36±0.03	0.032±0.003
96482	0.51±0.01	0.53±0.02	0.86±0.02	0.032±0.003	0.91±0.13	0.012±0.001
94662	0.28±0.01	0.37±0.04	1.02±0.07	0.065±0.01	0.81±0.13	0.018±0.004

**Table 3.** Parameters from mtanh() fits to the calculated $T_{e}$ profiles from the ETG model for the JET-ILW pulses discussed in §4: $T_{e,\mathrm{ped}}^{\mathrm{ETG}}$ at pedestal top and pedestal width $\Delta_{T_{e}}^{\mathrm{ETG}}$ in $\psi_{N}$, ratio of calculated and measured heights and widths, for cases with different assumed values for $\eta_{e,\mathrm{cr}}$, β and $T_{e,\mathrm{sep}}$ used for the calculation.

pulse	case	$T_{e,\mathrm{ped}}^{\mathrm{ETG}}$	$\Delta_{T_{e}}^{\mathrm{ETG}}$	$T_{e,\mathrm{ped}}^{\mathrm{ETG}}/T_{e,\mathrm{ped}}$	$\Delta_{T_{e}}^{\mathrm{ETG}}/\Delta_{T_{e}}$	α	$\eta_{e,\mathrm{cr}}$	β	$T_{e,\mathrm{sep}}$
#	–	(keV)	($\psi_{N}$)	(–)	(–)	(–)	(–)	(–)	(keV)
84794	A	0.90	0.037	0.97±0.05	0.65±0.12	0.85	1.28	1.43	0.1
87342	A	0.46	0.057	0.76±0.04	0.80±0.09	0.85	1.28	1.43	0.1
96482	A	0.51	0.023	0.59±0.02	0.79±0.08	0.85	1.28	1.43	0.1
96482	B	0.79	0.019	0.91±0.02	0.66±0.07	0.85	1.28	1.43	0.065
94662	A	1.37	0.034	1.33±0.1	0.65±0.07	0.85	1.28	1.43	0.1
94662	B	0.91	0.041	0.89±0.06	0.76±0.09	0.85	0.8	2.9	0.1

For the high-gas pulse #87342, $T_{e,\text{ped}}$ is somewhat underpredicted (×0.76), while the predicted $\Delta_{T_{e}}$ is closer to the measured value (×0.8) than for the low-gas pulse. Note that the observation that the actual temperature pedestal is considerably narrower than the density pedestal, i.e. $\Delta_{T_{e}}/\Delta_{n_{e}}∼0.57$ and ∼0.36 in both the low- and high-gas cases, respectively, is reproduced by the model.

Note that in the high-gas pulse #87342, the locally gyro-Bohm normalized electron heat flux $Q_{e}^{⋆}$ is a factor ∼2--4 larger than in the low-gas pulse #84794 (see figure 2*f*), as a consequence of the weaker $T_{e}$ gradient in the former driving a similar absolute electron heat flux. Note that in the steep gradient region of the pedestal $Q_{e}^{⋆}≲O(1)$, while at the pedestal top where $R/L_{T_{e}}$ is much weaker, $Q_{e}^{⋆}$ is up to an order of magnitude larger. It is discussed in ref. [8] that this difference might be explicable in terms of increasing anisotropy ($k_{r}/k_{y}<1$) of the ETG turbulence as this transitions from the slab to the toroidal branch at higher values of $\eta_{e}$.

### (b) 3.5 MA/3.3 T high-power, ITER-baseline scenario pulse

A more stringent test of the model is to apply it to a case from a pulse with quite different parameters as those for which the ETG heat flux scaling was determined, e.g. as offered by the high-power, 3.5 MA ITER-baseline scenario H-mode pulse #96482 with ∼34 MW of heating power [27,28]. The high fraction of power radiated by W impurities in this pulse $F_{\text{Rad}}∼0.5$ results in a loss power due to inter-ELM heat transport $P_{\text{sep}}^{\text{iELM}}∼11\;\mathrm{MW}$ after accounting for the ELM loss power of $\left\langle P_{\text{ELM}}\right\rangle ∼5.3\;\mathrm{MW}$, which is again determined using the method described in ref. [4].

As reported in ref. [16], nonlinear GK calculations of the pedestal heat transport have been performed for a similar high-power, JET-ILW 3 MA ITER-baseline scenario pulse #92432, the behaviour of which is also discussed in detail in ref. [4]. In this case, with the assumption of realistic dilution by Be impurities, ∼80% of the conducted loss power across the pedestal could be explained by ETG turbulence. Hence, in our calculations for #96482, we assume that all of the inter-ELM pedestal heat transport is conducted through the electron channel, i.e. $P_{e,\text{sep}}=P_{\text{sep}}^{\text{iELM}}$.

**Figure 3.**
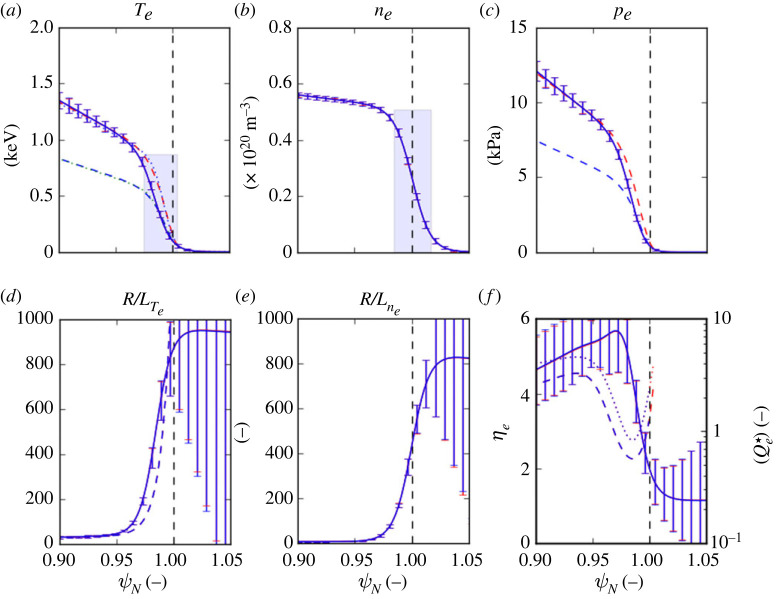
Pre-ELM averaged (∼80--100% of the inter-ELM period) pedestal profiles for the JET-ILW 3.5 MA/3.3 T ITER-baseline scenario pulse #96482 with 34 MW of heating power with deuterium gas fuelling and 35 Hz ELM pacing pellets. Two cases with the parameters stated in table 3 are shown using the nominal $Q_{e}^{⋆}$ scaling parameters A (cyan) with $T_{e,\text{sep}}∼100\;\mathrm{eV}$ and B (red) starting the integration where $T_{e}∼65\;\mathrm{eV}$. (Online version in colour.)

Pedestal profiles for this high-power 3.5 MA pulse #96482 are shown in figure 3. This pulse has a somewhat higher net fuelling rate from gas puffing and pellets $\Gamma_{D2}∼2.1\times 10^{22}\;e\;s^{-1}$ to that of the high-gas, 1.4 MA pulse #87342 ($∼1.8\times 10^{22}\;e\;s^{-1}$); however, the relative separatrix density $n_{e,\text{sep}}/n_{e,\text{ped}}∼0.4$ is not as high as in the latter pulse (∼0.6). This and the factor ∼2 higher loss power $P_{\text{sep}}^{\text{iELM}}$ results in a ∼×1.4 higher $T_{e,\text{ped}}∼0.86\;\mathrm{keV}$ than in the lower current, high gas pulse.

The most important difference between the pulses from the point of view of the predicted $T_{e,\text{ped}}$ is the value of toroidal field B, which is about twice as high in the 3.5 MA pulse than in the 1.4 MA pulses, i.e. 3.3 T c.f. 1.7 T. This reduces the gyro-Bohm normalization $q_{e,\text{gB}}\propto 1/B^{2}$ in the heat flux scaling equation (2.4) by a factor ×0.26, hence requiring a larger $R/L_{T_{e}}$ to match the prescribed heat flux $q_{e}$. In spite of this, the predicted $T_{e,\text{ped}}∼0.51\;\mathrm{keV}$ is a factor ∼0.6 below the actual value (case A in table 3), while the width $\Delta_{T_{e}}$ is better reproduced, namely, to a factor ∼0.8 of the actual value.

In §5, it is shown by means of an analytic model that a consequence of stiff ETG heat transport is a high sensitivity of the predicted $T_{e,\text{ped}}$ to the boundary conditions at the separatrix, in particular to the relative separatrix density $n_{e,\text{sep}}/n_{e,\text{ped}}$. Because of the steep gradient of $R/L_{n_{e}}$ at the separatrix, the predicted $T_{e,\text{ped}}$ is particularly sensitive to the separatrix location and uncertainties in the measured profiles. Reducing the assumed value of $T_{e,\text{sep}}$ from 100 eV to 65 eV decreases $n_{e,\text{sep}}$ (×0.78) and increases the initial value of $R/L_{n_{e}}$ (×1.6), consequently *increasing* the predicted $T_{e,\text{ped}}$, better matching the actual value (∼×0.93) (case B in table 3). Note that the two-point SOL model predicts that the separatrix temperature is a weak function of the loss power, i.e. $T_{e,\text{sep}}\propto P_{e,\text{sep}}^{2/7}$, so this is a rather large adjustment.^8^

Alternatively, the predicted $T_{e,\text{ped}}$ can also be increased by increasing the nonlinear threshold $\eta_{e,\text{cr}}$ from the nominal value of 1.28, e.g. to 2.4, matching $T_{e,\text{ped}}$ to a factor ×0.91. This purely conjectural change to the $Q_{e}^{⋆}$ scaling could only be confirmed by means of further nonlinear GK calculations. In all three cases, the normalized heat flux $Q_{e}^{⋆}$ shown in figure 3*f* is of similar magnitude and profile shape to that in the 1.4 MA pulses in figure 2.

## 5. Discussion

As discussed in §1, across the steep-density gradient region of the pedestal, the parameter $\eta_{e}$ is typically observed to be ∼O(2). It is illuminating to consider the consequences for the predicted $T_{e}$ profile of assuming: (i) that above a critical $\eta_{e,\text{cr}}$, the electron heat transport is infinitely stiff, i.e. clamping $\eta_{e}$ at this threshold, and (ii) that $\eta_{e,\text{cr}}$ is constant across the pedestal [4]. The definition of $\eta_{e}=L_{n_{e}}/L_{n_{e}}$ is actually a differential equation for $T_{e}$, i.e. $T_{e}^{\prime }=\eta_{e}T_{e}(n_{e}^{\prime }/n_{e})$, which for constant $\eta_{e,\text{cr}}$ can be integrated analytically inwards from the separatrix to yield:

5.1 $$T_{e}(\psi_{N})=T_{e,\text{sep}}{\left(\frac{n_{e}(\psi_{N})}{n_{e,\text{sep}}}\right)}^{\eta_{e,\text{cr}}}$$

which highlights the importance of the boundary conditions at the separatrix in determining $T_{e}$ across the pedestal if the heat transport is stiff. Referring to $T_{e}$ at the top of the density pedestal as $T_{e,\text{ped}}^{⋆}$, equation (5.1) then gives: $T_{e,\text{ped}}^{⋆}\equiv T_{e}(\psi_{N,n_{e,\text{ped}}})=T_{e,\text{sep}}{\left(n_{e,\text{ped}}/n_{e,\text{sep}}\right)}^{\eta_{e,\text{cr}}}$.

This relation implies that, if very stiff heat transport were to clamp $\eta_{e}$ to the critical threshold, $T_{e,\text{ped}}$ would then be: (i) highly sensitive to the relative separatrix density $n_{e,\text{sep}}/n_{e,\text{ped}}$; (ii) independent of the electron heat flux $q_{e}$ across the pedestal; and (iii) independent of the density pedestal width $\Delta_{n_{e}}$. Note that ref. [29] discusses the role of the relative separatrix density in governing the turbulent heat transport across the pedestal in JET-ILW. Also, in ref. [30], the effect of the relative shift $\delta_{n-T}$ on the MHD stability of the pedestal is investigated, showing that the reduced shift $\delta_{n-T}$ at low rates of gas puffing results in higher values of pedestal pressure $p_{e,\text{ped}}$.

Furthermore, it can be shown numerically that when $\eta_{e,\text{cr}}>1$ across the pedestal, the predicted $T_{e}$ profiles are shifted radially inwards with respect to the $n_{e}$ profiles, i.e. $\delta_{n-T}=\psi_{N,n_{e,\text{ped}}}-\psi_{N,T_{e,\text{ped}}}>0$, as is evident from the profiles shown in figures 2 and 3. Also, the predicted $T_{e}$ pedestal width $\Delta_{T_{e}}$ is narrower than that of the density $\Delta_{n_{e}}$ and vice versa for $\eta_{e}<1$. Of course, when $\eta_{e}=1,$ the shapes of the profiles are identical and $\delta_{n-T}=0$. The actual values of $\Delta_{T_{e}}$, $\Delta_{n_{e}}$, their ratios and relative shifts $\delta_{n-T}$ listed in table 2 qualitatively conform to this behaviour.

Average values of $\eta_{e}$ across the density pedestal ($\psi_{N,n_{e,\text{ped}}}<\psi_{N}<1$) stated in table 4 are in the range ${\left\langle \eta_{e}\right\rangle }_{\text{ped}}∼2\text{--}3$. Using these values of ${\left\langle \eta_{e}\right\rangle }_{\text{ped}}$ and the measured values of $n_{e,\text{ped}}/n_{e,\text{sep}}$ stated in table 4 in equation (5.1) yields values of $T_{e,\text{ped}}^{⋆}$, which are a factor ∼1.5 higher than the actual values of $T_{e}(\psi_{N}^{n,\text{top}})$. Interestingly, it has been found for a heating power scan over the range 4.6−16 MA, including the same 1.4 MA low-gas pulses as discussed in §4a, ${\left\langle \eta_{e}\right\rangle }_{\text{ped}}$ remains approximately constant across the steep-density region of the pedestal, while the increasing $T_{e,\text{ped}}$ with $P_{\text{in}}$ can at least partly be attributed to $n_{e,\text{sep}}$ decreasing approximately as $P_{e,\text{sep}}^{-1/2}$, even when taking into account the weak dependence of $T_{e,\text{sep}}$ on the loss power $T_{e,\text{sep}}\propto P_{e,\text{sep}}^{2/7}$.

**Table 4.** Parameters from the analytic model discussed in §5 for the JET-ILW pulses discussed in §4: $T_{e}$ at density pedestal top, average value of $\eta_{e}$ across the density pedestal, calculated value of $T_{e,\mathrm{ped}}^{⋆}$ using equation (5.1) and the ratio of this estimate to the measured values.

pulse	$T_{e}(\psi_{N}^{n,\mathrm{top}})$	${\left\langle \eta_{e}\right\rangle }_{\mathrm{ped}}$	$T_{e,\mathrm{ped}}^{⋆}$	$T_{e,\mathrm{ped}}^{⋆}/T_{e}(\psi_{N}^{n,\mathrm{top}})$
#	(keV)	(–)	(keV)	(–)
84794	0.57±0.06	1.9±0.1	0.84±0.3	1.5±0.6
87342	0.36±0.03	3.0±0.1	0.54±0.1	1.5±0.3
96482	0.45±0.03	3.1±0.2	0.67±0.14	1.5±0.3
94662	0.79±0.08	2.4±0.4	1.1±0.3	1.3±0.4

**Figure 4.**
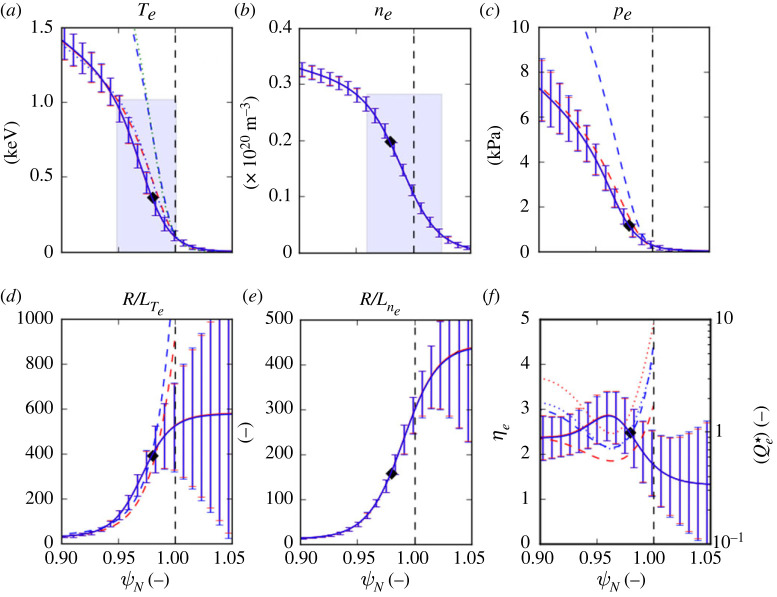
Pre-ELM averaged (80–100% of the inter-ELM period) pedestal profiles for the JET-ILW 3 MA ITER-baseline scenario pulse #94662 with 29 MW of heating power with zero rate of deuterium gas puffing. Predicted $T_{e}$ profiles (dashed) calculated using the nominal $Q_{e}^{⋆}$ scaling (cyan) and the stiffer scaling $Q_{e}^{⋆}=0.85{\left(\eta_{e}-0.8\right)}^{2.9}$ (red) are also shown. (Online version in colour.)

As shown in figure 4, when using the numerical model to predict the $T_{e}$ profile for a typical JET-ILW 3 MA pulse #94662 with 30 MW of heating and without gas puff fuelling during the sustained H-mode phase [31],^9^ using the nominal coefficients in the heat flux scaling determined for the 1.4 MA pulses, the resulting $T_{e}$ is too high (by a factor of ∼1.3) because of the high initial value of $R/L_{T_{e}}$ obtained at the separatrix from the $Q_{e}^{⋆}$ scaling.

For this ‘zero-gas’ pulse, the density pedestal is about twice as wide ($\Delta_{n_{e}}∼0.065$), while the relative separatrix density $n_{e,\text{sep}}/n_{e,\text{ped}}∼0.23$ is about half that as in the high-power 3.5 MA pulse #96482 shown in figure 3, which has a high rate of gas fuelling. The resulting low value of $n_{e}$ at the separatrix then requires high values of $R/L_{T_{e}}$ and $\eta_{e}$, which are much higher than the measured values, to conduct the prescribed electron heat flux $q_{e}$ across this region, while $\eta_{e}$ is actually approximately constant across the pedestal, with an average value ${\left\langle \eta_{e}\right\rangle }_{\text{ped}}∼2.4$.

By adopting a *stiffer* heat flux scaling of the form $Q_{e}^{⋆}\propto {\left(\eta_{e}-0.8\right)}^{2.9}$, i.e. with $\eta_{e,\text{cr}}$ reduced from 1.28 to the linear ETG threshold and increasing the exponent, the $T_{e}$ profile for this wide pedestal can be reproduced reasonably well, matching $T_{e,\text{ped}}$ to a factor ×0.89 and $\Delta_{T_{e}}$ to a factor ×0.76. Note that in this case, the alternative of shifting the profiles to higher $T_{e,\text{ped}}$ and hence *decrease* $R/L_{n_{e}}$ at the separatrix is unable to reduce $T_{e,\text{ped}}$ sufficiently unless a high value of ∼200 eV is assumed.

The resulting profiles from the full ETG model of §3 confirm the observation for all cases considered here that $R/L_{T_{e}}$ is overestimated outside the mid-pedestal and underestimated towards the pedestal top. This perhaps indicates that the actual electron heat transport is stiffer in the region just inside the separatrix but not as stiff towards the pedestal top as at the mid-pedestal location, particularly so for this low collisionality pedestal.

It is unlikely that the heat losses from the electrons directly from the pedestal region due to ionization and radiation are sufficient to significantly reduce $q_{e}$ close to the separatrix. In ref. [28], these power losses, estimated for the 3 MA JET-ILW baseline scenario pulse #92432 at 32 MW heating power with a similarly high-gas fuelling rate to that in pulse #96482, are shown to be relatively small (≲O(0.1 MW) and ≲O(1 MW) due to ionization and radiation, respectively) compared to the power conducted across the pedestal through the electron channel O (10 MW). In the lower current, lower power pulses, with lower gas fuelling rates discussed here, these losses are expected to be still less significant.

Taken together, these observations indicate that: (i) other turbulent modes, e.g. KBMs or MTMs, might contribute significantly to the electron heat flux in the region just inside the separatrix; and/or (ii) there may be other relevant parameters governing the scaling of the turbulent electron heat flux due to ETG turbulence, e.g. magnetic shear $\hat{s}$, which increases strongly close to the separatrix or perhaps the electron collisionality $\nu_{⋆,e}$. Note that the linear GENE calculations for the low-gas cases discussed in ref. [11] do show the presence of KBMs in the region just inside the separatrix.

## 6. Conclusion

By using the scaling of the locally, gyro-Bohm normalized heat flux $Q_{e}^{⋆}$ with $\eta_{e}$ found in ref. [11] to fit results of nonlinear GENE calculations of ETG turbulence for the steep gradient region of JET-ILW pedestals, it is possible to calculate the $T_{e}$ profile using the numerical model presented in §3 for the same 1.4 MA pulses for which the scaling was derived with reasonable agreement in terms of the profile shape, pedestal height $T_{e,\text{ped}}$ and width $\Delta_{T_{e}}$.

This model reproduces various observations and dependencies of the pedestal structure: the rather weak dependence of $T_{e,\text{ped}}$ on the heating power (at fixed $n_{e,\text{sep}}/n_{e,\text{ped}}$) and pedestal width $\Delta_{n_{e}}$; the different widths $\Delta_{n,T}$ of the $T_{e}$ and $n_{e}$ profiles; their relative shift $\delta_{n-T}$ and how these parameters and $T_{e,\text{ped}}$ depend on the relative separatrix density $n_{e,\text{sep}}/n_{e,\text{ped}}$. However, there are some obvious discrepancies, i.e. that this scaling overpredicts $R/L_{T_{e}}$ outside the mid-pedestal location and underpredicts $R/L_{T_{e}}$ towards the pedestal top, hence under predicting $\Delta_{T_{e}}$, although these differences partially compensate yielding a better estimate of $T_{e,\text{ped}}$.

Due to the strong dependence of the gyro-Bohm normalization of the $Q_{e}^{⋆}$ scaling on $B^{-2}$, $T_{e,\text{ped}}$ is predicted to increase with the toroidal field, approximately as $B_{t}^{2/3}$. The application of the model to a high-power 3.5 MA pulse with twice the toroidal field, significantly underestimated $T_{e,\text{ped}}$, which could be resolved either by assuming a lower value of $T_{e,\text{sep}}$, which reduces $n_{e,\text{sep}}$ or by an upshift of the threshold $\eta_{e,\text{cr}}$. An explanation of the sensitivity of $T_{e,\text{ped}}$ to the relative separatrix density $n_{e,\text{sep}}/n_{e,\text{ped}}$ is offered by a simple analytic model of infinitely stiff electron heat transport clamping $\eta_{e}$ to the critical threshold across the pedestal.

A further comparison for the case of a wide, low collisionality pedestal of a high-power pulse with zero rate of gas fuelling shows that overprediction of $R/L_{T_{e}}$ close to the separatrix using the nominal $Q_{e}^{⋆}$ scaling is propagated inwards by the integration, resulting in too high $T_{e}$ across the pedestal. However, the $T_{e}$ profile can be well predicted by a modified scaling with $\eta_{e,\text{cr}}$ reduced the linear ETG threshold and an increased stiffness exponent. This indicates that the ETG heat flux scaling may not be generally valid, so more work on investigating parametric dependence of the normalized electron heat flux $Q_{e}^{⋆}$ on other parameters, e.g. $\hat{s}/q=R/L_{s}$, …is required.

Such work currently being undertaken by the IFS group [32], attempting to determine a heat flux scaling to fit a database of nonlinear GENE turbulence simulations of pedestals from a variety of devices, hints that a stiffer scaling with $\eta_{e}$ may be a better fit to the gyro-Bohm normalized heat flux data. Of course, it may well be that other turbulent modes are involved in the electron heat transport, e.g. MTMs at the pedestal top or KBMs at the pedestal foot, so further detailed GK calculations are required to elucidate the underlying heat transport mechanisms.

Further comparisons of the model predictions with a wider range of pedestals are also required to determine the range of validity of the heat flux scaling used here, which is applicable when slab-ETG modes dominate the electron heat transport, both from JET-ILW and other devices, e.g. the MAST-U spherical tokamak. Previous GK micro-stability calculations, at ion scales in the pedestal region of MAST [22], found that KBM modes with twisting parity are the dominant ion scale modes in the steep pedestal region and that there is a transition to dominant tearing parity MTMs in the shallower pressure gradient region immediately inside the pedestal top.

As discussed in ref. [29], ITER is predicted to operate at a high ratio of separatrix to pedestal density $n_{e,\text{sep}}/n_{e,\text{ped}}≳0.4$ and at low pedestal collisionality [33], so the density gradient across the pedestal will be weaker than in the cases discussed here and with higher values of the parameter $\eta_{e}$. Under such conditions, toroidal ETG modes, which respond to a threshold in $R/L_{T_{e}}$ rather than $\eta_{e}$ [9], and/or MTMs are likely to be important contributors to the turbulent heat flux [11,15], so the model as formulated here may not be appropriate under such conditions.

Work is also underway to incorporate the numerical model of §3 for the $T_{e}$ profile into EPED. The current implementation assumes a given pedestal density $n_{e,\text{ped}}$, decreasing the width $\Delta_{n_{e}}$ until the MHD stability limit is reached, obviating the need to determine the pedestal width using the KBM constraint. This revised model predicts a very strong decrease of the pedestal width $\Delta_{p}$ on the relative separatrix density $n_{e,\text{sep}}/n_{e,\text{ped}}$, in contrast to the weak dependence predicted by the original EPED model. Further work is underway to compare these differing predictions with measurements. A complete prediction would also require a model for the density profile and for onset of particle and additional heat transport once the total pressure gradient exceeds the KBM stability limit.

## Data Availability

This article has no additional data.

## Footnotes

1 ELMs are explosive, edge-localized instabilities, which occur when the pedestal pressure reaches the MHD stability limit.

2 The normalized poloidal pressure is defined as: $\beta_{p}=2p_{e}/(2\mu_{0}{\bar{B}}_{p}^{2})$, where ${\bar{B}}_{p}$ is the flux surface averaged poloidal magnetic field.

3 Turbulent transport is considered stiff when the associated heat flux increases more strongly than linearly with the driving temperature gradient above that required to kindle the turbulence.

4 Note that charge-exchange reactions with cold neutral atoms is an energy loss from the ion channel, not the electrons. Also, as discussed in §5, power losses due to ionization and radiation directly from the pedestal region are relatively small compared to that conducted through the pedestal through the electron channel $P_{e,\text{cond}}$.

5 The effect of neglecting the fractional change in the flux surface area ($S\propto \psi_{N}$, where $\psi_{N}$ is the normalized poloidal flux enclosed by the surface) across the pedestal is small compared to other approximations made in the analysis, i.e. ΔS∼5% across the typical width of the pedestal $\Delta_{n_{e}}∼0.05$ expressed in terms of $\psi_{N}$.

6 The discriminant of a cubic polynomial is defined in terms of its coefficients as $\Delta =(4{\left(b^{2}-3ac\right)}^{3}-{\left(2b^{3}-9abc+27a^{2}d\right)}^{2})/27a^{2}$.

7 Note that it was found in ref. [4] that for the low-gas 1.4 MA pulses these loss power fractions were quite constant across the heating power scan.

8 The purpose of this speculative change to $T_{e,\text{sep}}$ is primarily to increase the value of $R/L_{n_{e}}$ at the separatrix, a quantity which has large measurement uncertainties due to its steep gradient.

9 Note that there is fuelling from neutral deuterium recycling in the divertor but no direct gas puffing.
